# Cracking Mechanism and Life-Cycle Performance Evaluation of Early-Age Concrete Based on Environment-Damage Coupling

**DOI:** 10.3390/ma19061256

**Published:** 2026-03-22

**Authors:** Min Yuan, Zhiqiang Xie, Jiazheng Li, Yun Dong, Sheng Qiang

**Affiliations:** 1 Changjiang River Scientific Research Institute, Wuhan 430010, China; xiezhiqiang@mail.crsri.cn (Z.X.); lijz@mail.crsri.cn (J.L.); dongyun@mail.crsri.cn (Y.D.); 2 National Center for Dam Safety Engineering Technology Research, Wuhan 430010, China; 3 College of Water Conservancy and Hydropower Engineering, Hohai University, Nanjing 210098, China; sqiang2118@hhu.edu.cn

**Keywords:** performance evaluation, high temperature, varying humidity, damage, strain, cracking mechanism

## Abstract

Concrete is accelerating its transition towards green and low-carbon development, but its performance throughout its entire life cycle is significantly influenced by environmental changes, which remains a key technical challenge currently faced. The effects of early-age concrete tensile damage on thermal conductivity and moisture transport properties, as well as their coupling mechanism, remain unclear, leading to severe cracking. To explore the cracking mechanism of early-age concrete under the coupled conditions of environment and damage and to evaluate its performance throughout its lifecycle, this article conducts comparative experiments on the performance of concrete under high temperature, varying humidity, and damage conditions in the early age stage. The variation law of temperature, humidity, and strain of concrete is studied, and the evolution of microstructure and composition of concrete is explored. The response of porosity to ambient humidity exhibits opposite trends between restrained and unrestrained specimens, with rates of change of +0.0353%/RH and −0.0245%/RH, respectively. Furthermore, the study identified a critical turning point in ambient relative humidity (50% RH), which significantly alters the degree of hydration (Ca/Si ratio) of the concrete. The research results may provide theoretical and technical support for cracking risk assessment and crack control throughout the entire life cycle of concrete thin-walled structures.

## 1. Introduction

Guided by the “dual carbon” strategy, the construction of water conservancy and hydropower projects is accelerating its transition towards a green and low-carbon development model. Compared with concrete dams, thin-walled structures in large-volume hydraulic concrete, such as the panels and gates of rockfill dams, are more susceptible to changes in environmental temperature [1,2] and humidity [3,4]. At present, extreme weather is becoming increasingly frequent, and some structural constraints are complex [5,6], which can easily cause damage to thin-walled concrete structures. However, concrete is most fragile at an early age and difficult to repair after damage [7,8], leading to a more common phenomenon of cracks during construction. With the widespread use of high-performance pumped concrete, the problem of cracks has become more prominent.

A large number of engineering practices have proven that 80% of concrete structural cracks are caused by non-load stresses caused by internal factors in concrete materials or due to changes in environmental temperature and humidity [9,10,11]. An increasing number of studies have shown that temperature and humidity deformation of concrete usually occur simultaneously, and the temperature and humidity fields inside concrete also interact with each other [12,13,14]. The change in humidity inside concrete does not exist independently but is largely influenced by factors such as temperature and stress. Especially under the cooperation of thermal and humidity coupling [15,16], the stress borne in the structure is greater, which is more likely to cause cracks, leading to material damage [17,18,19]. In turn, damage affects the temperature and humidity fields of concrete, and the possibility of cracks is increased by the coupling of damage, temperature, and humidity fields [20,21]. This seriously affects the safety of concrete structures. According to the micro mechanism, at the beginning of concrete tensile damage [21,22], the number of microcracks inside the concrete begins to increase. As the degree of damage increases, the number of microcracks gradually increases, and the humidity diffusion coefficient of the concrete will undergo a very significant change [23]. Currently, the understanding of the thermodynamic properties, moisture characteristics, and causes of cracking of concrete materials remains to be further explored [24], especially since the influence of early-age concrete tensile damage on heat and moisture transfer characteristics and its coupling mechanism are not yet clear, and the quantitative relationship between characteristic parameters and various factors at the macro and even micro levels still needs to be further studied. Furthermore, the service performance deterioration and failure of concrete structures [25,26] are essentially a progressive evolutionary process, where microscopic and mesoscopic damages continuously initiate and propagate, ultimately inducing the coalescence of macroscopic cracks. Therefore, conducting evaluations on the performance evolution laws throughout the full lifecycle of concrete, from initial damage incubation to cracking failure [27,28], holds significant theoretical value and engineering necessity for refining the durability design theories of concrete structures and ensuring their long-term service safety.

Therefore, in view of the current research status, where the cracking mechanisms of early-age concrete under the coupling conditions of high-temperature and variable-humidity environments and restraint-induced damage have not yet been fully elucidated, and the full-cycle evaluation system for its macro- and mesoscopic performance remains incomplete, this article conducted concrete parameter and performance experiments under high temperature, varying humidity, and damage conditions in the early age stage; studied and revealed the changes and phenomena of temperature, humidity, and strain of concrete; and explored the evolution of the microstructure and composition of concrete. The quantitative relationship between porosity and environmental temperature and humidity was obtained, and the turning point of environmental conditions affecting the degree of concrete hydration (Ca/Si) was discovered. By exploring the coupling mechanism of heat conduction and moisture transfer under tensile damage conditions in early age concrete, theoretical and technical support is provided for the cracking risk assessment and crack control of thin-walled structures in hydraulic large volume concrete during construction.

## 2. Experiment and Method

### 2.1. Mix Proportion of Concrete

According to the Chinese standard JGJ55-2011 [29], concrete specimens with C30 target strength grade were prepared, and the specific mix proportions are shown in Table 1 [22]. 

### 2.2. Experimental Design Method

The experimental environment control adopts a high and low temperature alternating humidity and heat test chamber, which measures a temperature range of −20 °C to 150 °C and a relative humidity range of 30% RH to 98% RH. Four typical working conditions under extreme environmental conditions are selected as representatives, each of which includes comparative experiments of non-restrained concrete specimens and restrained specimens. The specific experimental environmental conditions and purposes are shown in Table 2.

**Figure 1:**
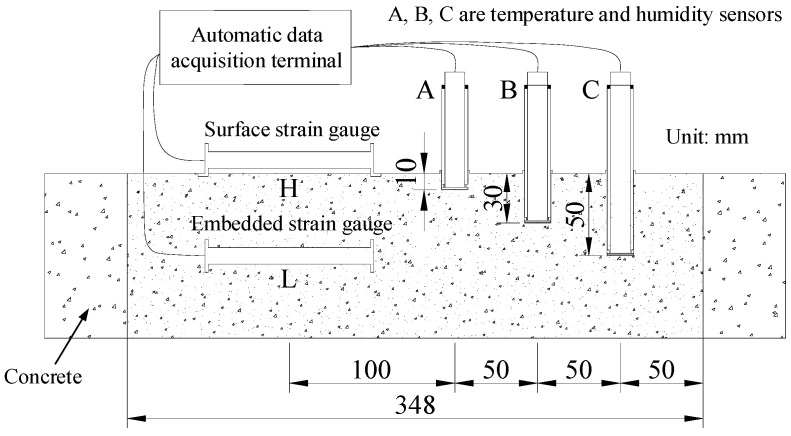
The buried position of the sensor.

**Figure 2:**
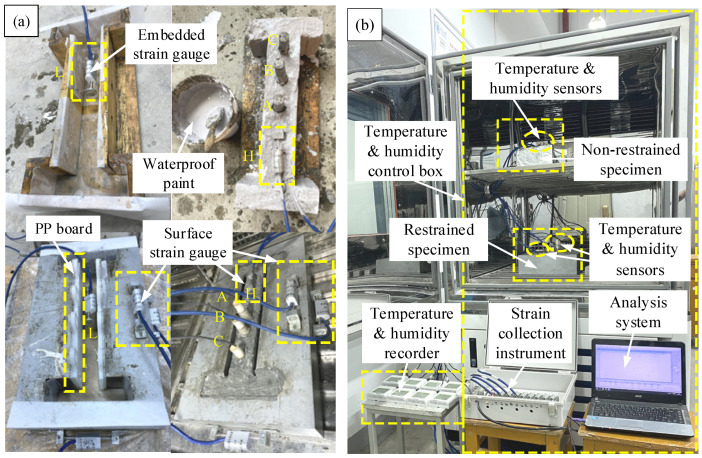
Schematic diagram of concrete specimen forming and experiment: (**a**) Forming of concrete specimens; (**b**) Overall overview.

The depth of temperature and humidity sensors in concrete is 1 cm, 3 cm, and 5 cm, corresponding to numbers A, B, and C, respectively. The strain gauge sensors are numbered L and H, respectively. Among them, the strain gauge sensors are of the vibrating wire type and have been calibrated before leaving the factory. The schematic diagram of the buried position of the sensor is shown in Figure 1. After the basic preparation work is ready, the experiment is started according to the test plan, with a loading time of 28 days and data recorded every 1 min. After 24 h, data is recorded every 5 min. The formation and overall schematic diagram of concrete specimens are shown in Figure 2.

### 2.3. Characterization of Microscopic Structures

**Figure 3:**
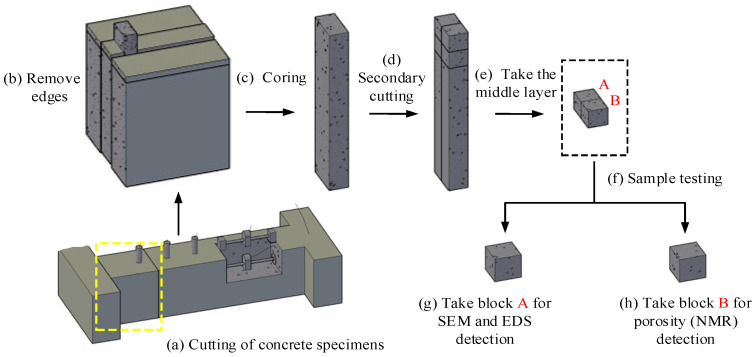
Sample preparation process for characterizing the microstructure of concrete: (**a**) cutting of concrete specimens; (**b**) removal of poorly exposed sides of the specimen; (**c**) select the core sample on the left side of the symmetry plane; (**d**) secondary cutting, select the upper core sample; (**e**) select the middle layer as the final core sample; (**f**) conduct relevant tests on the samples; (**g**) take block A for SEM and EDS detection; (**h**) take block B for porosity (NMR) detection.

To analyze the effects of environmental temperature and humidity on the performance of non-restrained and restrained concrete specimens, this experiment used testing methods such as SEM, EDS, and nuclear magnetic resonance (NMR) to study the microstructure morphology characteristics and composition evolution of concrete. After the end of the experiment (28th day of age), cut and sample the concrete specimens. The specific sample preparation process and purpose are shown in Figure 3. According to the experimental plan, when the ambient temperature is 65 °C and the humidity is 30%, it corresponds to one experiment. Therefore, the non-restrained specimen samples under constant temperature and humidity environmental conditions are numbered A1-1, the restrained specimen samples are numbered B1-1, and the other numbers are analogous. The specific numbers (areas) are shown in Table 3.

## 3. Experimental Results and Discussion

### 3.1. Changes in Temperature, Humidity, and Strain

**Figure 4:**
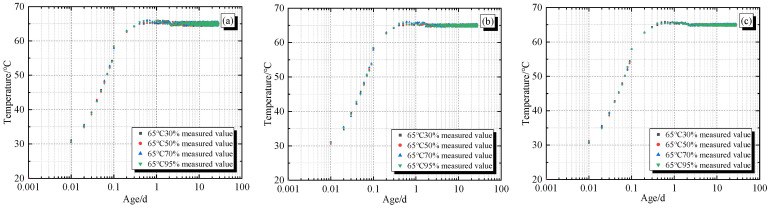

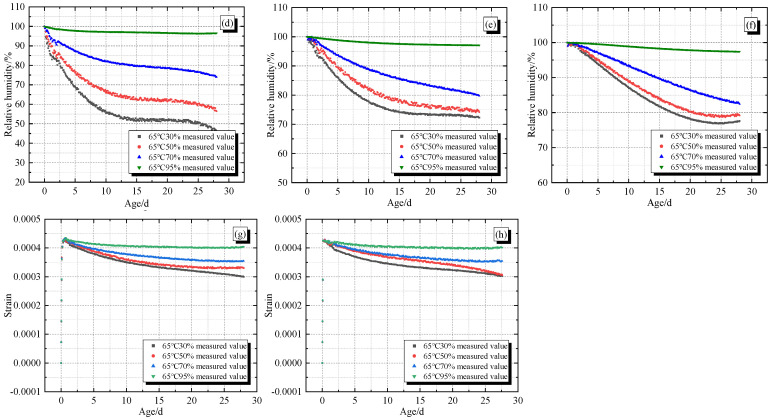
Duration curves of temperature, humidity, and strain for non-restrained specimens: (**a**) temperature of A; (**b**) temperature of B; (**c**) temperature of C; (**d**) relative humidity of A; (**e**) relative humidity of B; (**f**) relative humidity of C; (**g**) strain of L; (**h**) strain of H.

According to the number of sensors, the measured data of temperature, humidity, and strain of concrete specimens were organized. Figure 4a–c corresponds to the temperature duration curves of sensors A, B, and C (hereinafter referred to as A, B, and C) under different environmental conditions. Figure 4d–f correspond to the humidity duration curves of sensors A, B, and C, and Figure 4g,h correspond to the strain duration curves of sensors L and H (hereinafter referred to as L and H).

From Figure 4a–c, it can be seen that when the ambient temperature is 65 °C, the temperature of non-restrained concrete specimens A, B, and C under different environmental humidity conditions rapidly rises to their peak value in a short period of time and remains basically stable, with almost no difference. Under the same environmental humidity conditions, the temperature difference at different positions is not significant, mainly due to the small size of the concrete specimens themselves, which react quickly at higher environmental temperatures, and the environmental humidity does not have enough time to affect the concrete specimens in a short period of time.

As shown in Figure 4d, under the same environmental humidity conditions, the humidity of A decreases with age. Under different environmental humidity conditions, the lower the environmental humidity, the faster the humidity decrease rate of A, which is more obvious in the early age and tends to stabilize in the later stage. The main reason is that the dissipation of concrete moisture includes two effects: the hydration reaction of cementitious materials and moisture evaporation. In the early age stage, the hydration moisture consumption effect of cementitious materials dominates. When the environmental humidity is lower, it will promote the evaporation of surface moisture of concrete, leading to a more significant decrease in humidity of A. The humidity fluctuates slightly at around 21 days, and the lower the environmental humidity, the more significant the amplitude of the fluctuation. This may be due to the influence of damage detection, which leads to changes in the humidity inside the environmental box, causing changes in the measured values of the humidity probe. However, the overall trend of the measured values decreasing with age under different environmental conditions is almost consistent. From Figure 4d–f, it can be seen that due to B and C being further away from the upper surface, the humidity of B and C has overall increased relative to A.

As the distance between the measuring point and the surface depth increases, the environmental humidity decreases by the same magnitude, while the humidity of A, B, and C decreases by a decreasing trend. For example, under the conditions of environmental humidity of 30% and 50%, the humidity of A in 10 days is 55.577% and 65.929%, with a difference of 10.352% (the latter—the former, the same later), the humidity of B in 10 days is 77.900% and 82.195%, with a difference of 4.295%, and the humidity of C in 10 days is 87.066% and 88.997%, with a difference of 1.931%. It can be seen that the deeper the measurement point is from the upper surface, the less affected by the environment.

From Figure 4g,h, it can be seen that under different environmental humidity conditions, L and H are first subjected to thermal expansion, leading to a sudden increase in strain to the highest point. Then, due to the loss of moisture on the surface of the concrete, dry shrinkage occurs, and the strain gradually decreases with age, with similar trends and sizes of both. Taking L analysis as an example, the strain under different environmental humidity conditions reaches its peak at around 0.5 days. As the total strain actually includes temperature strain, dry shrinkage strain, elastic strain, self-generated volume deformation, creep, etc., the temperature suddenly rises significantly in a short period of time, with temperature strain accounting for the main part. For surface points, thermal expansion manifests as “tensile strain”. With the increase in age, the continuous loss of moisture on the concrete surface leads to dry shrinkage of the concrete surface, and dry shrinkage strain accounts for the main part, manifested as “compressive strain”. As the “compressive strain” gradually increases, the total strain gradually decreases. The lower the environmental humidity, the greater the rate and magnitude of total strain reduction. Also affected by damage detection, the strain fluctuates slightly around 21 days. The lower the environmental humidity, the more significant the amplitude of the fluctuation. However, the overall variation pattern of the measured values, decreasing with age under different environmental conditions, remains almost consistent.

**Figure 5:**
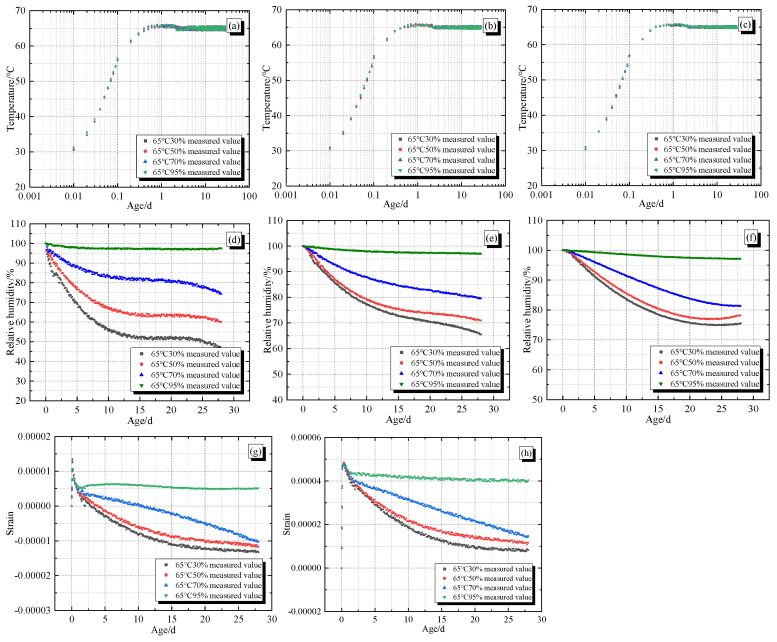
Duration curves of temperature, humidity, and strain for restrained specimens: (**a**) temperature of A; (**b**) temperature of B; (**c**) temperature of C; (**d**) relative humidity of A; (**e**) relative humidity of B; (**f**) relative humidity of C; (**g**) strain of L; (**h**) strain of H.

For concrete restrained specimens, as shown in Figure 5a–c, it can be seen that when the ambient temperature is 65 °C, the temperatures of A, B, and C under different environmental humidity conditions also rapidly rise to their peak values and remain stable, with almost no difference. Moreover, under the same environmental humidity conditions, the temperature differences at different points are not significant.

From Figure 5d–f, it can be seen that under the conditions of environmental humidity of 30% and 50%, the relative humidity decrease in restrained specimen A relative to non-restrained specimen is 0.120% and −0.777%, respectively. The relative humidity decrease in B relative to the non-restrained specimen is 0.350% and 3.064%, respectively. The relative humidity decrease in C relative to the non-restrained specimen is 3.343% and 3.466%, respectively. It can be seen that the constraint effect increases the damage to the concrete, thereby increasing the rate of moisture transfer from the inside to the surface, which in turn leads to a decrease in the humidity inside the concrete specimens (B, C).

Under the condition of environmental humidity of 50%, the decrease in humidity of A actually “increases”. The reason is that the damage accelerates the transfer of internal moisture to the surface. The higher the environmental humidity, the slower the relative loss of moisture, leading to the accumulation of moisture on the surface. The overall trend is that humidity does not decrease but increases instead. For example, compared with the non-restrained specimen, the restrained specimen showed a slight increase in overall humidity of A under different environmental humidity levels (30% → 95%) at an age of 21 days, with increases of −0.502%, 2.345%, 1.873%, and 0.627%, respectively.

From Figure 5g,h, it can be seen that under different environmental humidity conditions, L and H are also subjected to a sudden increase in thermal expansion strain to the highest point, and then dry shrinkage is caused by the loss of moisture on the surface of the concrete. The strain gradually decreases with the increase in age, and the trend and magnitude of the two changes are similar to those of L and H in non-restrained specimens, but the magnitude of the relative decrease in strain in non-restrained specimens is almost one level different. It can be seen that the significant constraint restricts the expansion of concrete specimens, leading to a significant decrease in strain.

### 3.2. SEM Microscopic Morphology

**Figure 6:**
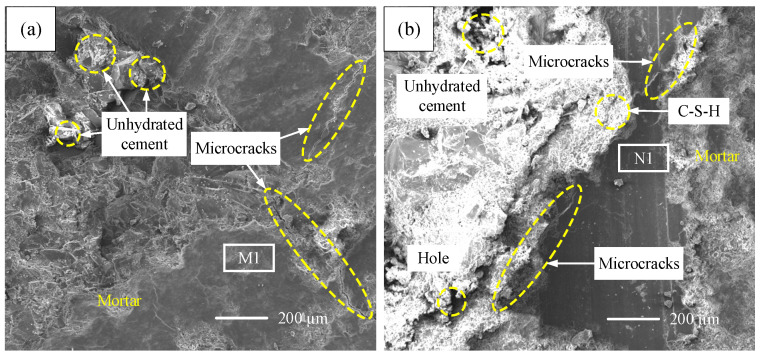

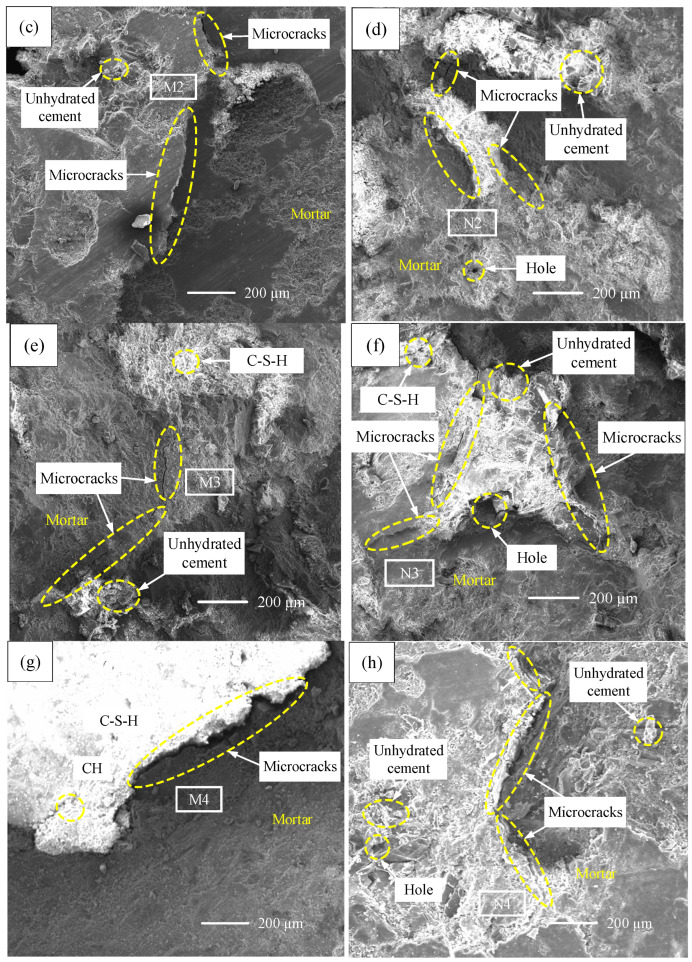
Comparison of microstructure in specimen samples: (**a**) A1-1 microstructure and region M1 position (65 °C 30%); (**b**) B1-1 microstructure and region N1 position (65 °C 30%); (**c**) A1-2 microstructure and region M2 position (65 °C 50%); (**d**) B1-2 microstructure and region N2 position (65 °C 50%); (**e**) A1-3 microstructure and region M3 position (65 °C 70%); (**f**) B1-3 microstructure and region N3 position (65 °C 70%); (**g**) A1-4 microstructure and region M4 position (65 °C 95%); (**h**) B1-4 microstructure and region N4 position (65 °C 95%).

According to the test results of the samples, as shown in Figure 6, concrete samples (A1-1~A1-4, B1-1~B1-4) under different humidity conditions of 65 °C all exhibit varying degrees of surface whitening, which is related to the uneven hydration of the cementitious material under high temperature conditions.

From Figure 6a,c,e,g, it can be seen that as the environmental humidity decreases (95% RH → 30% RH), the whitening phenomenon of non-restrained specimens A1-1 to A1-4 becomes less obvious, and the amount of unhydrated cement increases relatively. The degree of surface roughness is relatively more obvious. The reason is that although high-temperature environmental conditions can “promote” the hydration of cementitious materials, this “promotion” effect is uneven and insufficient. The hydration products are partially wrapped around the incomplete hydration part, causing an incomplete hydration reaction, and the decrease in environmental humidity will exacerbate this phenomenon. For example, in Figure 6a, the hydration product C-S-H of sample A1-1 under 30% environmental conditions at 65 °C is relatively less than that of sample A1-4 under 95% environmental conditions at 65 °C. The surface roughness of the sample is relatively obvious, and the density is relatively poor. In addition, samples A1-1 to A1-4 under different environmental humidity conditions have varying degrees of microcracks. The lower the environmental humidity, the more microcracks there are, which is mainly related to the accelerated surface moisture loss of concrete specimens under high temperature and low humidity conditions, leading to increased drying shrinkage.

From Figure 6b,d,f,h, it can be seen that as the environmental humidity decreases (95% RH → 30% RH), the whitening phenomenon and hydration product C-S-H of restrained specimens B1-1~B1-4 all slightly increase. The degree of surface roughness and the number of microcracks of the samples also slightly decrease, and the difference is not significant. This is opposite to the trend of changes in non-restrained specimens A1-1~A1-4, but there is still a lot of unhydrated cement. Moreover, the overall whitening phenomenon, the number and width of microcracks, the number of pores, and the degree of surface roughness of samples B1-1~B1-4 have increased compared to A1-1~A1-4, and the surface density has also decreased. It can be seen that under high temperature and low humidity environmental conditions, the constraint effect has a significant impact on the hydration of concrete cementitious materials and the generation of microcracks.

### 3.3. Analysis of EDS Spectroscopy

**Figure 7:**
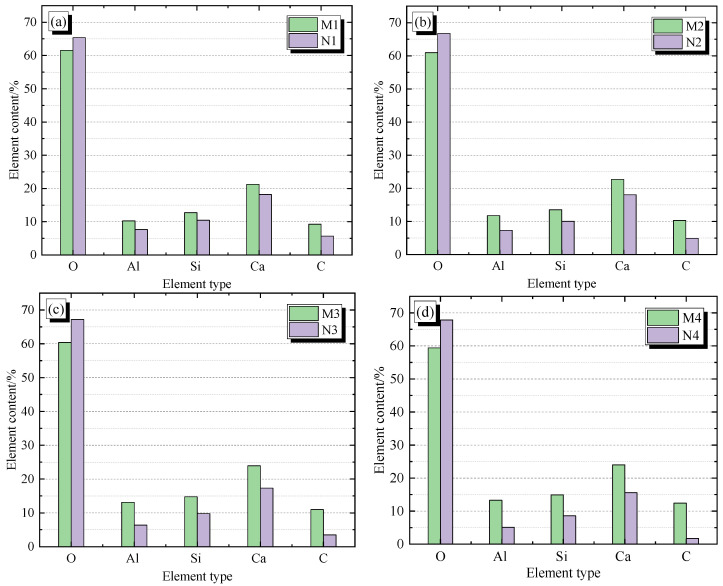
Comparison of EDS elements in different regions of specimen samples: (**a**) 65 °C 30%; (**b**) 65 °C 50%; (**c**) 65 °C 70%; (**d**) 65 °C 95%.

**Figure 8:**
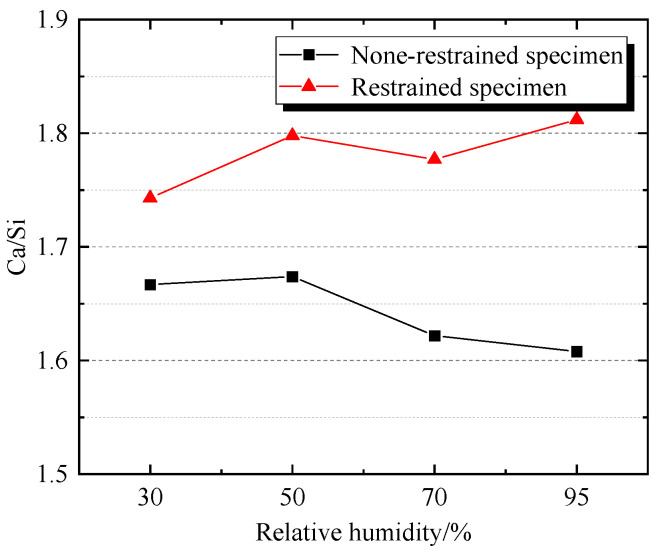
Comparison of Ca/Si in different regions of the specimen samples.

Research has shown that Ca/Si can be used as an evaluation criterion for the degree of concrete hydration crystallization [30,31]. EDS element analysis was conducted on concrete samples under different humidity conditions at 65 °C on the 28th day. The positions of each region were marked in the SEM samples, and the microcracks in the samples were selected as much as possible for each region. The EDS elements in each region and the results of Ca/Si were compared, as shown in Figure 7 and Figure 8.

From Figure 7a–d, it can be inferred that the hydration products of the sample areas M1 to M4 and N1 to N4 under different humidity environmental conditions at 65 °C are all composed of O, Ca, Si, Al, and C elements. It can be inferred that there are hydration products such as C-S-H, C-H, SiO_2_, Al_2_O_3_, and CaCO_3_ in all areas. In addition, except for the O element, the hydration products in areas M1 to M4 are smaller than those in areas N1 to N4, and all other elements are larger than those in areas N1 to N4.

From Figure 8, it can be seen that the Ca/Si values in the non-restrained specimen sample areas M1 to M4 are 1.667, 1.674, 1.622, and 1.608, respectively. Ca/Si values are all greater than 1.5, indicating that the overall trend of crystallinity increases with the increase in environmental humidity (30% RH → 95% RH). The Ca/Si values of the restrained specimen sample areas N1 to N4 are 1.743, 1.798, 1.777, and 1.812, respectively. Ca/Si values are also greater than 1.5, but this indicates that the overall trend of crystallinity decreases with the increase in environmental humidity. In addition, under different humidity environmental conditions at 65 °C, the Ca/Si of the sample areas M1~M4 were all smaller than those of the sample areas N1~N4, indicating that the crystallization of the restrained specimen samples was not as good as that of the non-restrained specimen samples. Moreover, when the environmental humidity was not higher than 50%, the Ca/Si of the non-restrained specimen sample areas M1~M4 changed less with the environmental humidity, and the Ca/Si of the restrained specimen sample areas N1~N4 changed more with the environmental humidity. Conversely, the Ca/Si of the restricted sample areas M1~M4 and N1~N4 showed opposite trends. “50% RH” may be the environmental humidity turning point that affects the degree of hydration crystallization (Ca/Si) of concrete specimens. The variation pattern of Ca/Si in regions M1 to M4 and N1 to N4 is generally consistent with the variation pattern of sample microstructure in Section 3.2.

It can be seen that the degree of crystallization of the sample is not only related to environmental temperature and humidity, but also to the constraint effect. Constraints can cause damage, which in turn exacerbates the generation of microcracks. Microcracks can lead to a decrease in the degree of hydration of the cementitious material, and the interrelationships between different factors are complex.

### 3.4. Analysis of Changes in Porosity

**Figure 9:**
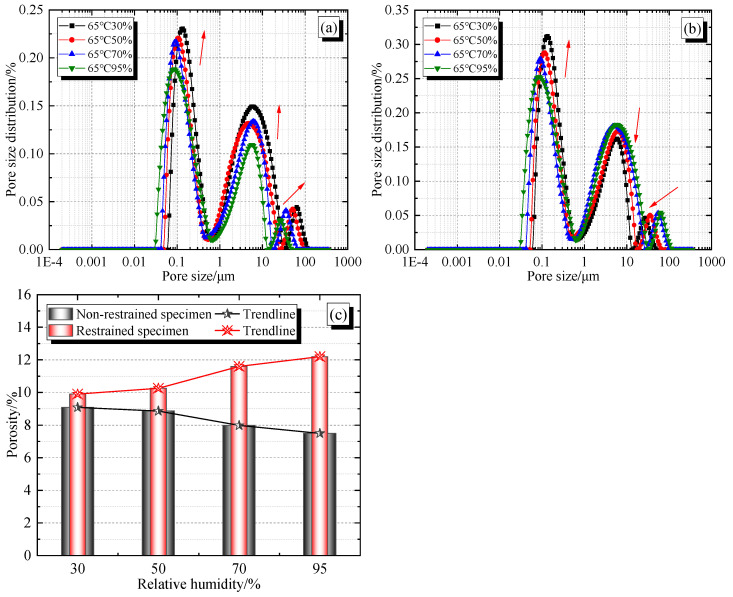
Comparison of pore size distribution and porosity in specimen samples: (**a**) Pore size distribution of non-restrained specimen samples; (**b**) Pore size distribution of restrained specimen samples; (**c**) Porosity of specimen samples.

According to the test results of the samples, as shown in Figure 9a,b, the pore size distribution of concrete samples (C1-1 to C1-4, D1-1 to D1-4) under different humidity environmental conditions at 65 °C basically shows a “three peak” shape (small pore, mesopore, and macropore).

From Figure 9a, it can be seen that for non-restrained specimens C1-1 to C1-4, as the environmental humidity decreases (→ direction), the pore size distribution curve shifts upwards to the right as a whole, and the peak rises rapidly, indicating a relative increase in internal pores and microcracks in the concrete. The reason is related to the decrease in the degree of hydration of the cementitious material caused by the low-humidity environment. With the change in environmental humidity (95% RH → 30% RH), the pore size distribution range ranges from 0.032 to 47.442 μm, and becomes 0.065~113.075 μm. The maximum and minimum apertures have increased by 65.633 μm and 0.033 μm, respectively. As shown in Figure 9c, the porosity increases with the decrease in environmental humidity (95% RH → 30% RH) (7.490% → 9.082%), an increase of 1.592%. The change rate of relative environmental humidity is −0.0245%/RH, indicating that high temperature and low humidity environmental conditions have a significant adverse effect on the pore size and porosity of concrete.

From Figure 9b, it can be seen that for the restrained specimens D1-1 to D1-4, as the environmental humidity decreases, the peak values of small pores also shift upwards to the right, but the peak values of medium and large pores shift downwards to the left. The porosity decreases with the decrease in humidity (95% RH → 30% RH) (12.200% → 9.903%), a decrease of 2.297% (the change rate of relative environmental humidity is +0.0353%/RH). On the other hand, with the change in humidity (95% RH → 30% RH), the pore size distribution range ranges from 0.034 to 121.562 μm, and becomes 0.070–51.003 μm. The maximum and minimum apertures have decreased and increased by 70.559 μm and 0.036 μm, respectively. It indicates that with the decrease in environmental humidity, the constraint effect has little effect on the pore size of small pores, and has an “inhibitory” effect on the pore size of medium and large pores. However, the overall distribution of medium and large pore sizes has a significant “increase” compared to the non-restrained specimen samples C1-1 to C1-4 under different humidity conditions of 65 °C, resulting in a relative increase of 4.399% (95% RH) and 0.821% (30% RH) in porosity compared to the non-restrained specimen samples C1-1 to C1-4 under different humidity conditions of 65 °C, as shown in Figure 9c. This also indicates that the constraint effect has a significant adverse effect on the pore size and porosity of concrete. In addition, when the environmental humidity is not higher than 50%, the porosity of concrete samples (C1-1~C1-4, D1-1~D1-4) changes less with environmental humidity, and vice versa, indicating that “50% RH” may be the environmental humidity turning point affecting the degree of hydration crystallization (Ca/Si) of concrete specimens.

## 4. Summary and Conclusions

This research adopts a combination of macroscopic mechanical experiments and microscopic inspections to explore the coupling mechanism of heat conduction and moisture transfer in early-age concrete under tensile damage conditions. The main findings and conclusions are as follows:

(1) The constraint effect will increase the tensile damage of concrete, thereby accelerating the transfer rate of internal moisture to the surface. The higher the environmental humidity, the slower the relative loss of moisture, leading to the accumulation of moisture on the surface and a decrease in internal humidity.
(2) The overall constraint effect will lead to an increase in the number and width of microcracks, the number of pores, and the degree of surface roughness, and a decrease in surface density. The reason is that high temperature environmental conditions can promote the hydration of cementitious materials, but this promotional effect is uneven and insufficient. The hydration products are partially wrapped around the incomplete hydration part, causing an incomplete hydration reaction. The decrease in environmental humidity will exacerbate this phenomenon.
(3) Environmental humidity exhibits opposite regulatory effects on the hydration product characteristics and porosity evolution of free and restrained specimens. As environmental humidity increases, the Ca/Si of free specimens decreases while the crystallinity of hydration products improves, and porosity shows a downward trend, with a rate of change of −0.0245%/RH relative to environmental humidity. In contrast, the Ca/Si of restrained specimens increases correspondingly, their crystallinity decreases, and porosity exhibits an upward trend, with a rate of change of +0.0353%/RH.
(4) Restraint significantly alters the hydration process and pore structure evolution characteristics of concrete, thereby increasing the Ca/Si of specimens and reducing the crystallinity of hydration products. At the pore structure level, restraint has a negligible effect on small pore sizes and exerts a certain “inhibitory” effect on the development of medium and large pores. However, it ultimately increases the overall pore size distribution across the entire range, leading to an increase in concrete porosity.
(5) An environmental humidity of 50% RH serves as a critical turning point affecting the degree of hydration crystallinity (Ca/Si) of concrete. Taking this as the boundary, the hydration process and Ca/Si evolution patterns of concrete exhibit significant differences. This humidity threshold is a core boundary condition for governing the hydration crystallization behavior of early-age concrete.

## Figures and Tables

**Table 1:** **Optimal** mix proportion of concrete [22].

Concrete Grade	w/c	Sand Content Rate	Water-Reducing Agent	Content/(kg·m^−3^)				
				Water	Cement	Sand	Aggregate I (5 to10 mm)	Aggregate II (10 to 25 mm)
C30	0.47	0.419	2.1	175	370	780	324	756

**Table 2:** Grouping of concrete specimens.

Specimen Type	Environmental Temperature Range/°C	Environmental Humidity Range/%	Purpose of the Experimental Specimens
Non-restrained specimen	65	30, 50, 70, 95	Temperature, humidity, strain, SEM, EDS, porosity
Restrained specimen	65	30, 50, 70, 95	Temperature, humidity, strain, SEM, EDS, porosity

**Table 3:** Number of concrete specimen samples (regions).

Category and Number	Constant Temperature and Humidity Environment (30%RH → 95%RH)	
	Non-Restrained Specimen Samples	Restrained Specimen Samples
SEM	A1-1, A1-2, A1-3, A1-4	B1-1, B1-2, B1-3, B1-4
EDS (regions)	M1, M2, M3, M4	N1, N2, N3, N4
Porosity (NMR)	C1-1, C1-2, C1-3, C1-4	D1-1, D1-2, D1-3, D1-4

## Data Availability

The original contributions presented in this study are included in the article. Further inquiries can be directed to the corresponding author.
